# Precision Modulation of Neurodegenerative Disease-Related Gene Expression in Human iPSC-Derived Neurons

**DOI:** 10.1038/srep28420

**Published:** 2016-06-24

**Authors:** Sabrina Mahalia Heman-Ackah, Andrew Roger Bassett, Matthew John Andrew Wood

**Affiliations:** 1 Department of Physiology, Anatomy and Genetics, University of Oxford, Oxford, OX1 3QX, UK; 2 NIH Oxford-Cambridge Scholars Program, National Institutes of Health, Bethesda, MD, 20892, US; 3 UNC MD-PhD Program, University of North Carolina at Chapel Hill, Chapel Hill, NC, 27599, US; 4 Genome Engineering Oxford, Sir William Dunn School of Pathology, University of Oxford, Oxford, OX1 3RE, UK

## Abstract

The ability to reprogram adult somatic cells into induced pluripotent stem cells (iPSCs) and the subsequent development of protocols for their differentiation into disease-relevant cell types have enabled in-depth molecular analyses of multiple disease states as hitherto impossible. Neurons differentiated from patient-specific iPSCs provide a means to recapitulate molecular phenotypes of neurodegenerative diseases *in vitro*. However, it remains challenging to conduct precise manipulations of gene expression in iPSC-derived neurons towards modeling complex human neurological diseases. The application of CRISPR/Cas9 to mammalian systems is revolutionizing the utilization of genome editing technologies in the study of molecular contributors to the pathogenesis of numerous diseases. Here, we demonstrate that CRISPRa and CRISPRi can be used to exert precise modulations of endogenous gene expression in fate-committed iPSC-derived neurons. This highlights CRISPRa/i as a major technical advancement in accessible tools for evaluating the specific contributions of critical neurodegenerative disease-related genes to neuropathogenesis.

Parkinson’s disease (PD) is a progressive neurodegenerative disease which manifests primarily as a devastating movement disorder. Alpha-synuclein, encoded by the SNCA gene, is perhaps the most commonly implicated molecular mediator of PD pathogenesis. Point mutations^1^,^2^,^3^ and multiplications^4^,^5^,^6^,^7^ of the SNCA gene, which promote aggregation of the alpha-synuclein protein, have been described as the causes of some familial forms of PD. Notably, although only a small portion of all PD cases are familial in etiology, alpha-synuclein pathology is similarly an important mediator of sporadic forms of the disease^8^,^9^,^10^. In addition to its role as a primary mediator of PD, alpha-synuclein pathology has been described as a common feature of other synucleinopathies, including PD with dementia (PDD), dementia with Lewy bodies (DLB) and multiple systems atrophy (MSA)^11^,^12^.

Comorbidity is often observed between the synucleinopathies and other neurodegenerative diseases which share the common molecular events of protein misfolding or accumulation, aggregation and deposition, collectively known as proteinopathies^13^. For example, overabundance of amyloid precursor protein, encoded by the APP gene, leads to its aggregation into β-amyloid, a well-established mechanism of Alzheimer’s disease (AD) pathology^14^,^15^. Overabundance of hyperphosphorylated microtubule-associated protein tau, encoded by the MAPT gene, is the central molecular pathogenic event of the tauopathies, of which AD^16^ and frontotemporal dementia with Parkinsonism linked to tau mutations on chromosome 17 (TDP-17)^17^ are notable examples. In addition, expanded CAG repeats in the HTT gene form aggregation-prone polyglutamine tracts in the huntingtin protein, a causative factor in Huntington’s disease (HD)^18^. The co-occurrence of features of synucleinopathy, amyloidopathy, tauopathy and polyglutaminopathy in cases that have been attributed purely to one disease classification has spurred the concept of a continuum between these diseases^13^,^19^. Thus, the development of novel tools for interrogating genetic contributions to the common processes underlying proteinopathies could result in therapies that benefit patients across a broad spectrum of neurodegenerative disorders^13^,^20^.

Since their discovery in 2006^21^, induced pluripotent stem cells (iPSCs) have been well recognized for their potential applications in regenerative medicine and disease modeling. iPSCs are adult somatic cells which have been reprogrammed to a pluripotent state using defined genetic^21^ or chemical factors^22^. Human iPSCs^23^ can be derived from patients with specific diseases^24^, allowing investigators unprecedented opportunities to probe the molecular mechanisms underlying the pathogenesis of diseases in which the affected cell type is inaccessible in living patients. The first studies to reveal the utility of iPSCs in modeling diseases of the nervous system demonstrated that disease-specific phenotypes can be observed in iPSC-derived neurons from patients with familial dysautonomia^25^ and spinal muscular atrophy (SMA)^26^. iPSCs have subsequently been used in several studies that have yielded important insights into the molecular underpinnings of neurodegenerative diseases^27^,^28^,^29^,^30^. Although iPSCs have demonstrated value in elucidating disease-relevant phenotypes that have not been gleaned from previous models, it remains challenging to perform precise alterations of neurodegenerative disease-related gene expression in iPSC-derived neurons – a technique which would further enhance the utility of iPSC models for characterizing the specific contributions of neurodegenerative disease-related genes to neuropathogenesis. Recent advances in genome editing technologies have revolutionized such efforts in mammalian systems, presenting novel methods for transcriptional modulation of disease-relevant gene expression in the modeling of complex human diseases.

The *Streptococcus pyogenes* CRISPR/Cas9 type II endonuclease system^31^ has recently been adapted for editing of the human genome^32^,^33^,^34^. Whereas wild-type Cas9 mediates DNA double-strand breaks, mutations of Cas9 have been discovered which render the endonuclease capable of cleaving only a single strand of DNA^35^,^36^ or abolish its endonuclease activity altogether^37^,^38^. We endeavored to explore the utility of nuclease null, or dead, Cas9 (dCas9) for transcriptional repression of the aforementioned proteinopathy-related genes. The D10A^−^/H840A^−^ dCas9 mutant was used to induce CRISPRi-mediated repression of SNCA, MAPT, HTT and APP via targeting to the transcription start sites (TSSs) of these genes. In addition to presenting data in support of the position dependence of dCas9 for efficient CRISPRi, specifically proximity to the TSS, we provide evidence that the affinity of the dCas9:DNA interaction, as mediated by sgRNA binding, is mechanistically critical to the success of transcriptional repression by CRISPRi. This data contributes to the growing body of knowledge regarding mechanistic determinants of CRISPRi efficacy which may eventually aid the rational design of dCas9 sgRNAs for robust gene silencing by CRISPRi^37^,^39^. Finally, we demonstrate that dCas9-KRAB^38^ and dCas9-VPR^40^ effector domain fusions can be used to exert precise alterations in the expression of the critical PD-related gene, SNCA, in human iPSC-derived neurons. The ability to execute precise transcriptional control over disease-associated gene expression in iPSC-derived neurons using dCas9 will further aid efforts to dissect molecular contributors to neurodegenerative diseases and provides an unprecedented opportunity to evaluate the temporal course of pathogenic events following neurodegenerative disease-related gene activation and repression.

## Results

### TSS Proximity and sgRNA-Mediated dCas9:DNA Binding Affinity are Critical Determinants of CRISPRi

**Figure 1:**
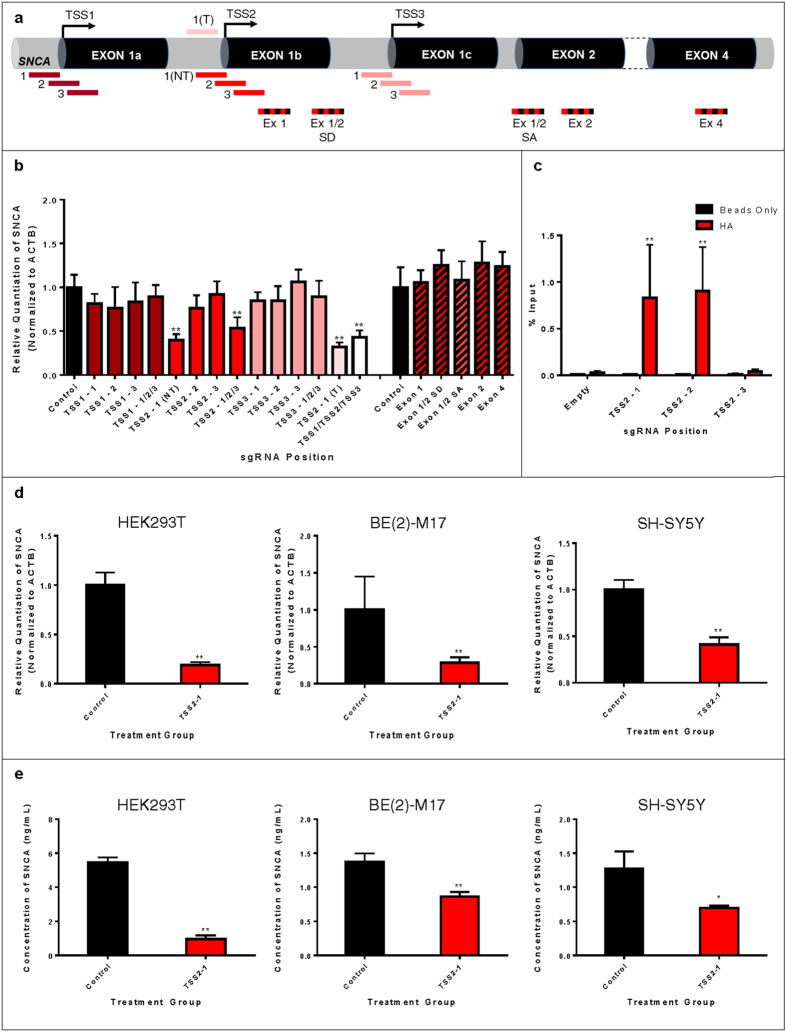
Screening of sgRNAs for CRISPRi Targeting SNCA Exons and Transcription Start Sites. (**a**) Schematic representation of SNCA exon- and TSS-targeting sgRNA positions. (**b**) qRT-PCR screening of total alpha-synuclein mRNA in HEK293T cells transfected with SNCA exon- and TSS-targeting sgRNAs and dCas9. Data are represented as mean ± SEM. (**c**) HA ChIP of HEK293T cells transfected with empty dCas9 vector or TSS2-1, TSS2-2, or TSS2-3 sgRNA co-expressing dCas9 vectors. Data are represented as mean ± SEM. (**d**) qRT-PCR for total alpha-synuclein mRNA in HEK293T, BE(2)-M17 and SH-SY5Y cells transfected with TSS2-1 sgRNA co-expressing dCas9 vector. Data are represented as mean ± SEM. (**e**) ELISA for alpha-synuclein protein in HEK293T, BE(2)-M17 and SH-SY5Y cells transfected with TSS2-1 sgRNA co-expressing dCas9 vector. Data are represented as mean ± SEM. *p ≤ 0.05 compared to control, **p ≤ 0.01 compared to control. SD = splice donor, SA = splice acceptor.

As alpha-synuclein is well-validated as a central mediator of PD pathogenesis^1^,^2^,^3^,^4^,^5^,^6^,^7^,^8^,^9^,^10^, we first evaluated the utility of dCas9 in executing transcriptional regulation of neurodegenerative disease-related genes by targeting the SNCA gene. SNCA is a six exon gene, wherein alternative splicing of exons 3 and 5 produces four transcript isoforms. As CRISPRi was previously shown to inhibit transcription elongation in *E. coli* and mammalian cells^37^, we tested the possibility of inducing SNCA gene repression by designing sgRNAs to target transcribed regions within exons expressed in all alpha-synuclein transcript isoforms, including exons 1, 2, 4, and the exon 1/2 splice donor (SD) and splice acceptor (SA) sites (Fig. 1a,b). However, no observable reduction in alpha-synuclein expression occurred using any of these sgRNAs.

**Figure 2:**
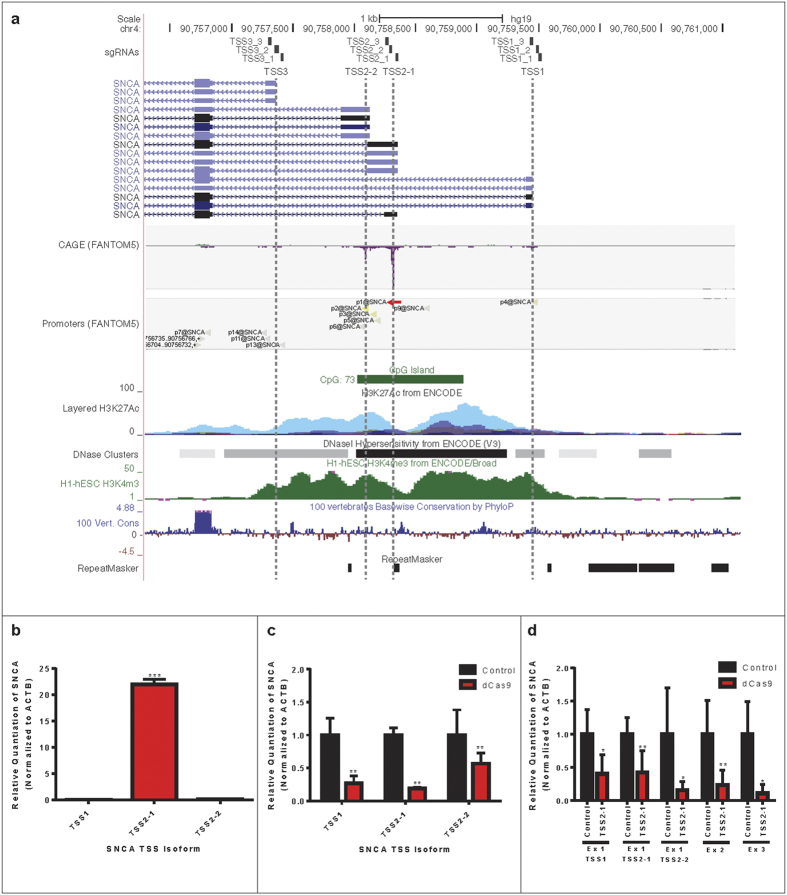
Effect of SNCA TSS2-1 sgRNA-Mediated CRISPRi on TSS Isoform-Specific mRNA Expression Levels. (**a**) SNCA promoter region. Predicted transcripts (from UCSC Genome Browser) and transcription start sites (TSS) are indicated, along with the position of the TSS-targeting sgRNAs (sgRNAs). Cap analysis of gene expression (CAGE) data from the FANTOM5 consortium shows the relative TSS usage, averaged across all samples, and positions of predicted promoter regions (Promoters, p1-14@SNCA). The promoter associated chromatin signatures, H3K27Ac, H3K4me3 and DNaseI hypersensitivity, show data from the Encyclopedia of DNA Elements (ENCODE) project. Basewise conservation (PhyloP) and repeats (RepeatMasker) are also shown. (**b**) qRT-PCR analysis of TSS usage in HEK293T cells demonstrates that TSS2-1 is the predominant TSS. The TSS3 transcript isoform was undetectable. Data are represented as mean ± SEM. (**c**) Expression of TSS isoform-specific mRNAs following TSS2-1 sgRNA-mediated CRISPRi. Analysis of expression levels of total SNCA mRNA and spliced transcripts specific to TSS1, TSS2-1 and TSS2-2 by qRT-PCR in dCas9/TSS2-1 sgRNA-transfected HEK293T cells (red bars, dCas9) relative to controls (black bars, control). Data are represented as mean ± SEM. (**d**) Analysis of nascent transcript expression following TSS2-1 sgRNA-mediated CRISPRi. Analysis of nascent transcripts was conducted on dCas9/TSS2-1 sgRNA transfected HEK293T cells (red bars, dCas9) relative to controls (black bars, control). Expression of nascent transcripts was estimated by qRT-PCR using primers spanning the exon 1:intron boundaries specific to transcripts deriving from TSS1, TSS2-1, and TSS2-2 or at the exon 2:intron and exon 3:intron boundaries. Data are represented as mean ± SEM. *p ≤ 0.05 compared to control, **p ≤ 0.01 compared to control, ***p ≤ 0.001 compared to control.

We next tested whether CRISPRi could induce efficient silencing of SNCA via inhibition of transcription initiation. Alpha-synuclein is expressed from at least four different promoters (Fig. 2a) based on EST sequences, cap analysis of gene expression (CAGE) from the FANTOM5 consortium^41^,^42^ and annotation in the Eukaryotic Promoter Database (EPDnew)^43^. We designed sgRNAs to the three TSSs annotated in EPDnew (human version 003). Specifically, sgRNAs were designed to align directly over each TSS, immediately upstream or immediately downstream on the non-template (NT) strand (Fig. 1a). We screened these sgRNAs for repressive activity in HEK293T cells and found the TSS2-1 (NT) sgRNA (henceforth TSS2-1) to have strong repressive activity when transfected alone, or within any combination of SNCA TSS-targeting sgRNAs (Fig. 1b). This is consistent with analysis of CAGE tags (Fig. 2a) and our own qRT-PCR data (Fig. 2b), which suggest that in HEK293T cells, transcription initiation is predominantly driven from this TSS. Analysis of CAGE tags from other tissues further indicates that SNCA transcription initiation in human brain tissue, neural stem cells and neurons is primarily from this TSS (Supplementary Table S3). Interestingly, despite sgRNA targeting to only one annotated TSS, the TSS2-1 sgRNA induced efficient silencing of spliced transcripts derived from all SNCA TSSs (Fig. 2c). Similar silencing of all TSS isoforms was observed when we analyzed nascent transcription by qRT-PCR with primers spanning specific exon-intron boundaries (Fig. 2d), suggesting coupling of expression from the three TSSs. The repressive effect on nascent transcripts further indicates that silencing by CRISPRi occurs at the level of transcription initiation, or the transition from initiation to elongation, rather than any post-transcriptional effect on the mRNA.

As dCas9 has been described to have superior repressive activity when targeting the non-template strand of a gene^38^, we tested whether an sgRNA aligning to the template (T) strand at the same SNCA TSS2-1 locus would induce silencing. Conveniently, the sequence of this guide allows a perfectly complementary sgRNA, TSS2-1 (T), to be designed to target an identical region on the template strand. Interestingly, the sgRNA aligning to the same position on the template strand was capable of inducing comparable silencing of alpha-synuclein transcript levels to the sgRNA on the non-template strand (Fig. 1a,b). This further suggests that the mechanism of repression may not involve steric hindrance of RNA polymerase II elongation, as postulated previously^38^, and instead may involve inhibition of transcription initiation.

As the repressive effects of dCas9 were observed in the absence of an effector domain fusion, we sought to define mechanistic determinants of CRISPRi in the absence of a transcriptional repressor. We first evaluated whether sgRNA-mediated dCas9:DNA binding affinity could explain the differences in repressive activity of different sgRNAs targeting the same TSS. As dCas9 is expressed with an HA tag, we used an anti-HA antibody to perform chromatin immunoprecipitation (ChIP) of dCas9 from HEK293T cells transfected with an empty dCas9 vector, lacking sgRNA expression, or a dCas9 vector co-expressing SNCA TSS2-1, TSS2-2 or TSS2-3 sgRNAs (Fig. 1c). TSS2-1, the sgRNA which induced the most robust gene repression, and TSS2-2, a weakly active sgRNA, showed enrichment at the SNCA genomic locus encompassing all three sgRNAs. In contrast, TSS2-3, an inactive sgRNA, was not enriched. This suggests that differences in binding affinity between different sgRNAs as well as their position relative to the TSS are both critical for efficient CRISPRi.

The SNCA TSS2-1 sgRNA was capable of reducing alpha-synuclein transcript (Fig. 1d) and protein (Fig. 1e) levels across multiple cell lines, including HEK293T, as well as the PD-relevant dopaminergic neuroblastoma cellular models, BE(2)-M17 and SH-SY5Y, examined by qRT-PCR and ELISA, respectively.

### Multiplex Transcriptional Repression of Neurodegenerative Disease-Related Gene Expression using CRISPRi

**Figure 3:**
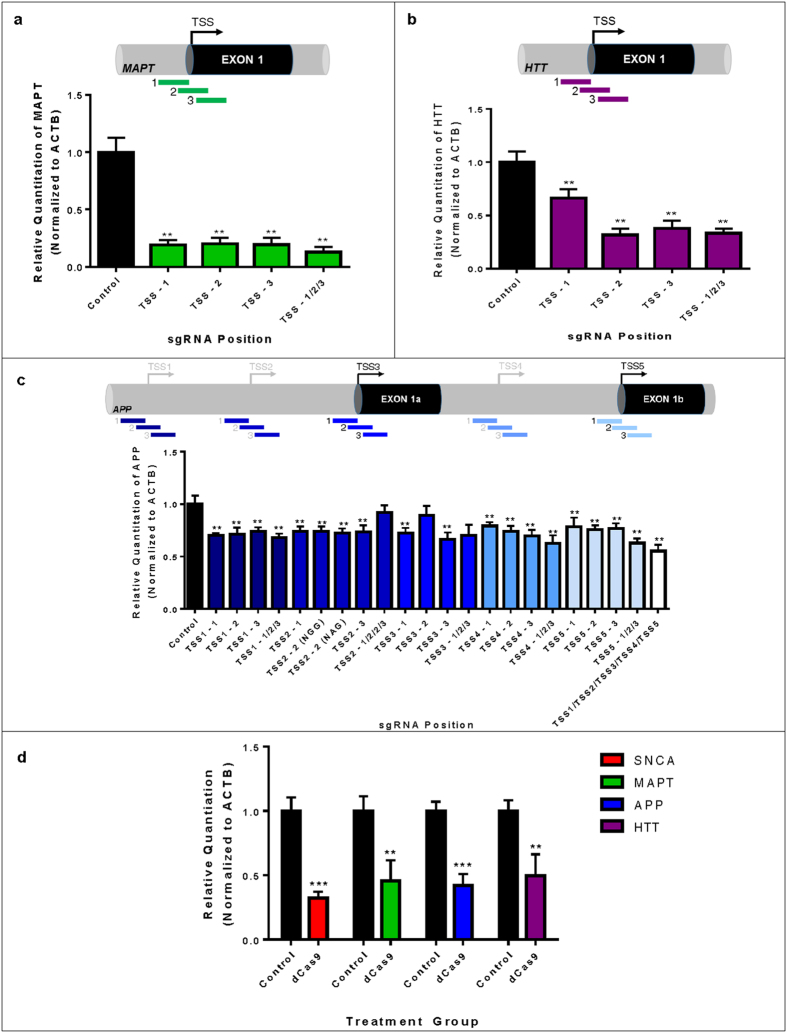
Multiplex Transcriptional Repression of Neurodegenerative Disease-Related Gene Expression Using CRISPRi. (**a**) qRT-PCR for microtubule-associated protein tau mRNA in HEK293T cells transfected with dCas9 sgRNAs targeting the MAPT TSS. Data are represented as mean ± SEM. (**b**) qRT-PCR for huntingtin mRNA in HEK293T cells transfected with dCas9 sgRNAs targeting the HTT TSS. Data are represented as mean ± SEM. (**c**) qRT-PCR screening of amyloid precursor protein mRNA in HEK293T cells transfected with dCas9 sgRNAs targeting the five annotated APP TSSs. TSSs represented in black were identified in both EPDnew and RefSeqGene, whereas those represented in grey are putative annotated TSSs in EPDnew. Data are represented as mean ± SEM. (**d**) qRT-PCR quantification of mRNA levels for alpha-synuclein, microtubule associated protein tau, amyloid precursor protein and huntingtin in HEK293T cells simultaneously transfected with the most active TSS-targeting dCas9 sgRNAs for SNCA, MAPT, APP and HTT. Data are represented as mean ± SEM. **p ≤ 0.01 compared to control, ***p ≤ 0.001 compared to control.

We next examined whether TSS-targeting of dCas9 could be applied to silence other genes critically involved in neurodegenerative diseases, including the genes most commonly implicated in Huntington’s (HTT)^18^ and Alzheimer’s (MAPT and APP)^14^,^15^,^16^ diseases. Congruent with the design of sgRNAs to target SNCA (described above), we designed sgRNAs to target the one annotated TSSs of MAPT (Fig. 3a) and HTT (Fig. 3b), and the five annotated TSSs of APP (Fig. 3c) as defined in EPDnew. Silencing of all three genes was achievable using one or a combination of TSS-targeting sgRNAs. Finally, we examined the possibility of targeting SNCA, MAPT, HTT and APP in the same cells by co-transfecting the most robust sgRNAs for each gene as identified by screening, and were able to induce repression of all of the targeted genes in the same cells at the same time (Fig. 3d). This demonstrates the feasibility of using dCas9 to perform complex manipulations of gene expression profiles to probe the contributions of specific genes, and combinations thereof, to neurodegenerative disease phenotypes.

### Precise Transcriptional Modulation of Alpha-Synuclein Expression in Human iPSC-Derived Neurons Using CRISPRa and CRISPRi

**Figure 4:**
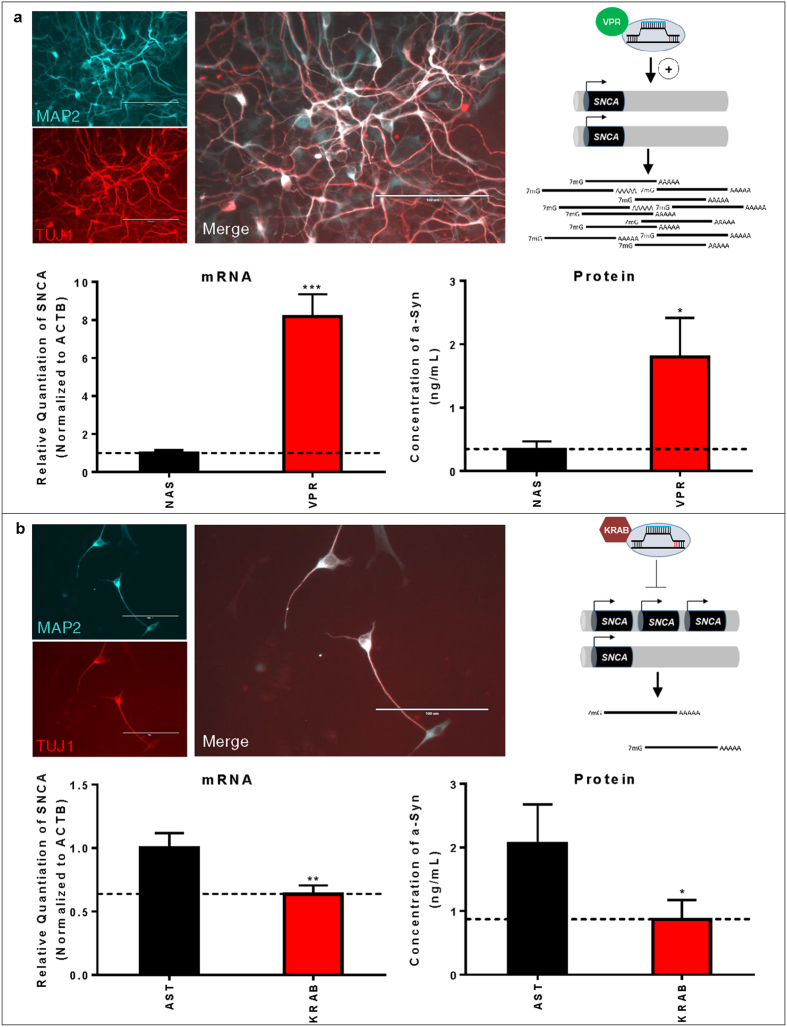
CRISPRa- and CRISPRi-Mediated Modulation of Alpha-Synuclein Transcript and Protein Expression Levels in Human iPSC-Derived Neurons. (**a**) CRISPRa-mediated activation of alpha-synuclein in NAS iPSC-derived neurons with TSS2-2 sgRNA and dCas9-VPR transcriptional activator. mRNA levels were quantified by qRT-PCR in biological triplicate, and data are represented as mean ± SEM. Protein levels were quantified by ELISA in biological triplicate, and data are represented as mean ± SEM. (**b**) CRISPRi-mediated repression of alpha-synuclein in AST iPSC-derived neurons with TSS2-1 sgRNA and dCas9-KRAB transcriptional repressor. mRNA levels were quantified by qRT-PCR in biological triplicate, and data are represented as mean ± SEM. Protein levels were quantified by ELISA in biological triplicate, and data are represented as mean ± SEM. Micrographs demonstrate the neuronal identity of iPSC derivatives as confirmed by co-immunostaining with the pan-neuronal markers, MAP2 and TUJ1 (scalebars are 100 μm). Schematic diagrams represent the intended outcome of CRISPRa/i-mediated gene expression modulation. *p ≤ 0.05 compared to control, **p ≤ 0.01 compared to control, ***p ≤ 0.001 compared to control. NAS = normal alpha synuclein; AST = alpha-synuclein triplication.

To further explore the utility of dCas9-mediated transcriptional alterations for interrogating the functions of neurodegenerative disease-related genes, we examined the possibility of using dCas9-effector domain fusions to exert precise alterations of gene expression in iPSC-derived neurons. NCRM-5 iPSCs were derived from a healthy control patient and express normal alpha-synuclein (NAS) levels. ND34391G iPSCs were derived from a patient with PD caused by alpha-synuclein triplication (AST). iPSCs were first quality controlled by morphological analysis, pluripotency immunostaining, karyotyping, TaqMan hPSC Scorecard analysis, and CNV quantification to confirm the genomic presence of two SNCA copies in NCRM-5 iPSCs and four SNCA copies in ND34391G iPSCs (Supplementary Fig. S1). iPSCs were then differentiated into a stable intermediate neural stem cell (NSC) population (Supplementary Fig. S2) via dual SMAD inhibition^44^. iPSC-derived neurons were differentiated from NSCs and neuronal identity confirmed by the expression of markers of fate-committed neurons using RT-PCR (Supplementary Fig. S2) and immunostaining (Fig. 4a,b). On day 7 of differentiation, neurons were Neon transfected in triplicate with dCas9-VPR/TSS2-2 sgRNA, dCas9-KRAB/TSS2-1 sgRNA, or a CAG-driven tdTomato transfection control plasmid (Supplementary Fig. S2).

Using the transcriptional activator, dCas9-VPR^40^ and TSS2-2 sgRNA (which bound with high affinity, but did not induce gene silencing), we were able to induce an 8-fold activation of endogenous SNCA expression in NAS iPSC-derived neurons, a level approximately equivalent to the expression of alpha-synuclein in AST neurons (Fig. 4a). Alpha-synuclein protein levels were quantified by ELISA and were similarly found to be upregulated approximately 5-fold in dCas9-VPR/TSS2-2 sgRNA-transfected neurons (VPR) compared to non-transfected controls (NAS) (Fig. 4a). In parallel, we demonstrated that the transcriptional repressor, dCas9-KRAB^38^ and TSS2-1 sgRNA, can be used to exert a 40% reduction in alpha-synuclein mRNA levels in AST iPSC-derived neurons (Fig. 4b). Likewise, alpha-synuclein protein levels were reduced by approximately 60% in dCas9-KRAB/TSS2-1 sgRNA-transfected neurons (KRAB) compared to non-transfected controls (AST) (Fig. 4b). These results highlight the promise of dCas9-mediated CRISPRa and CRISPRi for modeling complex neurodegenerative diseases and evaluating the specific contributions of disease-associated loci by allowing precise modulation of gene expression in human iPSC-derived neurons.

## Discussion

CRISPRa/i are gaining increasing attention as powerful tools for precise transcriptional modulation of endogenous gene expression. Since the discovery of this system in 2013^37^, coupling of dCas9 to repressive domains has been used to enhance CRISPRi in eukaryotes^38^. However, our results demonstrate that even in the absence of repressive domains, CRISPRi represents a robust system for transcriptional silencing of eukaryotic genes in a position-dependent manner, specifically, when proximal to the TSS of a gene. Similar position dependence for efficient CRISPRi has recently been observed at other human genes, suggesting that this may be a general phenomenon^39^. We show that the effect of CRISPRi is mediated by reduction in nascent transcription. Contrary to our expectations, we see silencing of transcription from all TSSs when only one is targeted. This could be explained by coupling between promoters by mechanisms such as inhibition of common transcription factor binding^45^, shared transcription factory usage^46^, reduced affinity of enhancer-promoter loops^47^, or reduced efficiency or directionality of transcription re-initiation driven by mRNA looping^48^. The remarkable activity of one sgRNA to induce transcriptional silencing from multiple TSSs of the same gene implies that robust therapeutic silencing of overexpressed pathogenic genes is potentially attainable with this approach.

We have also shown that sgRNA-mediated dCas9 binding affinity is a critical factor for the repressive activity of dCas9 in CRISPRi. This may be due to differences in the inherent binding affinity of individual sgRNAs, or chromatin structure around these regions. Consistent with this notion, recent data suggests that Cas9 can bind more effectively to sequences within DNase hypersensitive sites that are associated with promoter regions^49^. These findings regarding the mechanism of CRISPRi will be useful in the rational design of sgRNAs for CRISPRi, and may eventually aid in defining new algorithms to predict activity of CRISPRi at particular genomic loci.

We employed TSS-targeting via CRISPRi to induce multiplex silencing of genes involved in proteinopathy-induced neurodegeneration, further confirming that the targeting of CRISPRi to the TSS can be broadly applied and supporting the notion that simultaneous targeting of multiple genes involved in common pathological processes can be accomplished with CRISPRi. Importantly, we have demonstrated that both CRISPRa and CRISPRi can be used to execute precise transcriptional modulation of neurodegenerative disease-related genes in human iPSC-derived neurons. Such a technique will allow investigations of the temporal series of events resulting from manipulation of disease-related gene expression levels. This will enable evaluation of the order of molecular changes that result in particular phenotypic outcomes, both during disease progression and upon therapeutic intervention. In addition to the applicability of this approach for studying genes which are commonly implicated in the pathogenesis of neurodegenerative diseases, such an approach could potentially be used to examine the influence of particular pathways in these diseases, by perturbation of one or multiple pathway components^50^.

CRISPRa/i provide a valuable approach to conducting experimental alterations of gene expression. Both have similar efficacy to other methods for gene activation and repression, while possessing the comparable advantages of ease of design, cost effectiveness and manipulations of genes from their endogenous genomic contexts. CRISPRa/i would be predicted to elicit reduced off-target effects since Cas9 can tolerate fewer mismatches within the sgRNA sequence^51^,^52^,^53^ and additionally requires the presence of a protospacer adjacent motif (PAM)^38^. Although it appears that binding of dCas9 is more promiscuous than cleavage activity^49^,^54^ and requires a shorter region of complementarity with the sgRNA^55^, the specificity of repression elicited by dCas9 can be very high^56^. Our data and that of others^39^ suggest that only those sgRNAs targeting in close proximity to a TSS will elicit changes in gene expression, which reduces the chances of off target effects considerably. With growing knowledge of the mechanism by which CRISPRa/i can be induced efficiently and specifically, these tools can be expanded to dissect the specific influence of genes with putative roles in other neurodegenerative diseases such as C9orf72 in ALS/frontotemporal dementia (FTD) and LRRK2 in PD as well as in the development of therapies for such diseases. The application of CRISPRa/i to iPSC models will further extend the utility of both systems for modeling complex diseases and may aid in the development of novel CRISPR/Cas9-based therapeutic approaches.

## Methods

### HEK293T, SH-SY5Y and BE(2)-M17 Cell Culture and Transfection

HEK293T and SH-SY5Y cells were maintained in DMEM (Life Technologies) supplemented with 10% HyClone FBS (GE Healthcare). BE(2)-M17 cells were maintained in OptiMEM (Life Technologies) supplemented with 10% HyClone FBS. Medium was changed every two days and cells were passaged when 70% confluent. HEK293T and BE(2)-M17 cells were seeded at a density of 1.3 × 10^5^ cells per well to 24-well plates 24 hours before transfection with Lipofectamine 2000 (Life Technologies) according to the manufacturer’s protocol. Cells were transfected in triplicate with 500 ng final DNA used per well. SH-SY5Y cells were Neon (Life Technologies) transfected at a density of 1 × 10^6^ cells per reaction according to the manufacturer’s protocol, using default system settings (1400 V, 20 ms, 1 pulse). Cells were transfected in triplicate with 2 μg final DNA used per reaction. A CAG-driven tdTomato expression vector was used as a transfection control for all experiments.

### Differentiation and Transfection of iPSC-Derived Neurons

NCRM-5 iPSCs, derived from a healthy control subject with normal alpha-synuclein (NAS) expression levels, were obtained from the NIH Center for Regenerative Medicine (NIH CRM) and are distributed through RUDCR Infinite Biologics at Rutgers University. ND34391G iPSCs, derived from a patient with PD caused by alpha-synuclein triplication (AST), were obtained from the Coriell Institute and are distributed through the NINDS Repository Fibroblasts and iPSCs Collection. NCRM-5 and ND34391G iPSCs were quality controlled by comparison to the following established iPSC lines: NCRM-1 iPSCs, derived from a healthy control subject, obtained from the NIH CRM and distributed through RUDCR Infinite Biologics at Rutgers University; NHDF-1 iPSCs, derived from a healthy control subject and obtained from the James Martin Stem Cell Facility; ND38477C iPSCs, derived from a patient with PD caused by a compound heterozygous 255A and exon 3–4 deletion in the PARK2 gene, obtained from the Coriell Institute and distributed through the NINDS Repository Fibroblasts and iPSCs Collection (Supplementary Fig. S1). Normal alpha-synuclein (NAS) and alpha-synuclein triplication (AST) neurons were differentiated from NCRM-5 and ND34391G iPSCs, respectively, via a stable intermediate iPSC-derived NSC population (Supplementary Fig. S2). On day 0, 7.5 × 10^6^ NSCs were seeded onto poly-L-ornithine (Sigma)/laminin (Life Technologies)-coated T75 tissue culture flasks. On day 1, 100% medium was replaced with Neuronal Differentiation Medium consisting of Neurobasal (Life Technologies), 1X B27 supplement (Life Technologies), 1X GlutaMAX (Life Technologies), 20 ng/mL BDNF (R&D Systems) and 20 ng/mL GDNF (R&D Systems). 100% medium was replaced every other day. On day 7, neuronal progenitors were dissociated with Accutase (Life Technologies). Neuronal progenitors were Neon transfected at a density of 5 × 10^5^ cells per reaction with dCas9 and sgRNA expression plasmids according the manufacturer’s protocol, using default system settings (1400 V, 20 ms, 1 pulse). Neuronal progenitors were transfected in triplicate with 2 μg final DNA used per reaction. A CAG-driven tdTomato expression vector was used as a transfection control for all experiments (Supplementary Fig. S2). Following Neon transfection, cells were plated directly onto poly-ornithine/laminin-coated 12-well tissue culture dishes in Neuronal Differentiation Medium containing 10 μM ROCK inhibitor (Tocris Bioscience) to promote survival and 0.5 mM dbcAMP (Sigma) to promote maturation. Neurons were processed for analysis 72 hours after transfection. Neuronal identity was characterized by immunostaining for the pan-neuronal markers MAP2 and TUJ1 (Fig. 4a,b), as described below, and RT-PCR for markers of fate-committed neurons, including MAP2, TUJ1, NGN2, doublecortin, and neurofilament (Supplementary Fig. S2), as described below. All RT-PCR primers are listed in Supplementary Table S2.

### CRISPRi and CRISPRa Vector Design and Construction

CRISPRi constructs were generated from the pAC154-dual-dCas9VP160-sgExpression vector, which was a gift from Rudolf Jaenisch (Addgene #48240)^57^, by replacement of the VP160 domain with a puromycin acetyl transferase gene, with or without substitution of the sgRNA backbone, as described below. sgRNAs for inhibition of transcript elongation were designed to align at SNCA exon 1, 2, 4, and the exon 1/2 slice donor (SD) and splice acceptor (SA), and were expressed from a dCas9 vector with an unmodified sgRNA backbone. Constructs for TSS targeting of SNCA, HTT, MAPT and APP were designed to overlap the TSS, or to align immediately upstream or downstream of the TSS without overlapping. For CRISPRi, TSS-targeting sgRNAs were co-expressed in a dCas9 vector containing a modified F + E sgRNA backbone, where F + E refers to two modifications of the sgRNA backbone: F, an A – U basepair flip which abolishes a putative RNA polymerase III terminator (4 consecutive U’s) in the sgRNA stem-loop, and E, an extended dCas9-binding hairpin structure^58^. sgRNA oligos were annealed and subsequently ligated into dCas9 or dCas9 F + E vectors digested with *Bbs* I (NEB). For CRISPRa, SNCA TSS2-2 sgRNA and modified F + E sgRNA backbone were cloned with a human U6 promoter by overlap PCR into pGEM-Teasy (Promega) and co-transfected with the SP-dCas9-VPR vector, which was a gift from George Church (Addgene #63798)^40^. All sgRNA oligonucleotides are listed in Supplementary Table S1.

### RNA Extraction, Reverse Transcription, RT-PCR and qRT-PCR

Total RNA was extracted using the miRNeasy Mini Kit (Qiagen). cDNA was reverse transcribed from 1 μg of HEK293T and BE(2)-M17, 500 ng of SH-SY5Y, 350 ng of NAS iPSC-derived neuron, and 250 ng of AST iPSC-derived neuron RNA, using SuperScriptIII First-Strand Synthesis SuperMix for qRT-PCR (Life Technologies), per the manufacturer’s instructions. RT-PCR was performed using undiluted cDNA and Platinum PCR SuperMix (Life Technologies), per the manufacturer’s instructions. For qRT-PCR, HEK293T and BE(2)-M17 cDNA was diluted 1:200, SH-SY5Y cDNA was diluted 1:100, NAS iPSC-derived neuron cDNA was diluted 1:70, and AST iPSC-derived neuron cDNA was diluted 1:50 in nuclease-free water (Ambion). For nascent transcript analysis, RNA was DNase treated prior to qRT-PCR using the TURBO DNA-free Kit (Ambion), per the manufacturer’s instructions. qRT-PCRs were run with standard cycling using KiCqStart SYBR Green Master Mix (Sigma). All RT-PCR and qRT-PCR primers are listed in Supplementary Table S2.

### Quantification of Alpha-Synuclein Protein by ELISA

HEK293T, BE(2)-M17 and SH-SY5Y cells, as well as iPSC-derived neurons were collected in Cell Extraction Buffer (Life Technologies), supplemented with PMSF (Sigma) and protease and phosphatase inhibitors (Thermo Scientific) 72 hours after transfection. ELISA was performed using the Human Alpha-Synuclein ELISA Kit (KHB0061, Life Technologies), following the manufacturer’s instructions. Briefly, samples and Alpha-Synuclein Standards were prepared in Sample Diluent Buffer and added to Alpha-Synuclein Antibody-coated assay wells in duplicate. Alpha-Synuclein Detection Antibody was added to assay wells and plates were incubated at room temperature for three hours. Assay wells were washed four times with 1X Wash Buffer and Anti-Rabbit IgG HRP was added to each well. Plates were incubated for 30 min at room temperature. Assay wells were washed 4 times with 1X Wash Buffer and Stabilized Chromogen was added. Wells were incubated for 30 min at room temperature, after which an equal volume of Stop Solution was added and absorbance was read at 450 nm. A standard curve was created in GraphPad Prism 6 (GraphPad Software) and the program was used to calculate the unknown concentrations of alpha-synuclein in cell lysates.

### HA ChIP

HEK293T cells were seeded at a density of 1 × 10^6^ cells to 6-well tissue culture dishes. Cells were transfected in triplicate with Lipofectamine 2000 using 2500 ng DNA per well. After 48 hours, cells were harvested by rinsing once with cold PBS, applying TrypLE (Life Technologies) until cells detached, and breaking into single cells by trituration with equal volume DMEM supplemented with 10% HyClone FBS. Cells were counted and 2.2 × 10^6^ cells were processed for sonication. Cells were pelleted by centrifugation at 1300 rpm for 5 min. Cells were washed once with 500 μL of cold PBS. Supernatant was discarded and cells were resuspended in 500 μL of fresh cold PBS. Cross-linking was initiated by the addition of 13.5 μL of 35.6 – 38% formaldehyde (Sigma) for 10 min at room temperature. Subsequently, 57 μL of 1.25 M glycine was added for 5 min at room temperature to quench the reaction. A volume of 143 μL complete Buffer B (Diagenode) containing protease inhibitor (Diagenode) was added and samples were incubated for 5 min on ice. Chromatin was sheared using a Bioruptor (Diagenode) with high setting for 3 rounds of 10 cycles, each 30 sec on, 30 sec off, after which 957 μL complete Buffer A (Diagenode) containing protease inhibitors (Diagenode) was added to dilute SDS. An aliquot of chromatin from 200,000 cells (100 μL) was analyzed for shearing efficiency by phenol:chloroform extraction and ethanol precipitation and products visualized on a 1.5% agarose gel. Insoluble material was removed by centrifugation at 16,000 × g for 15 min at room temperature, and soluble chromatin pre-cleared with 40 μL protein A agarose (Millipore) for 1 hour. A sample of 40 μL was taken as 10% input, 400 μL for mock ChIP using protein A beads alone and 400 μL for HA-Cas9 ChIP with a rat monoclonal anti-HA antibody (3F10 affinity matrix 11815016001, Roche). Samples were incubated overnight at 4 °C with rotation, and washed once in low salt wash buffer (0.1% SDS, 1% Triton X-100, 2 mM EDTA, 150 mM NaCl, 20 mM Tris-HCl pH 8.0), once in high salt wash buffer (0.1% SDS, 1% Triton X-100, 2 mM EDTA, 500 mM NaCl, 20 mM Tris-HCl pH 8.0), once in lithium chloride wash buffer (0.25 M LiCl, 1% NP-40, 1% Na-Deoxycholate, 1 mM EDTA-NaOH pH 8.0, 10 mM Tris-HCl pH 8.0) and twice in TE buffer (10 mM Tris-HCl pH 8.0, 1 mM EDTA). Chromatin was eluted from the beads with 100 μL elution buffer (1% SDS, 0.1 M NaHCO_3_) for 5 min at 65 °C and 30 min at 25 °C, and crosslinking reversed overnight at 65 °C alongside the input samples. DNA was purified after digestion with 100 μg/ml RNase A for 1.5 hours at 42 °C and 200 μg/ml proteinase K for 1 hour at 45 °C using ChIP DNA Clean and Concentrator Columns (Zymo). DNA was analyzed by qPCR and results are represented as % input. Primer sequences are listed in Supplementary Table S2.

### Immunostaining

For immunostaining, cells were fixed with 4% paraformaldehyde diluted in 1X PBS for 15 min. Fixative was removed and cells rinsed three times with 1X PBS for 5 min each wash. Cells were then blocked with a solution of 0.3% Triton X-100 and 5% goat serum in 1X PBS for one hour at room temperature. Blocking reagent was aspirated and primary antibodies were applied in antibody dilution buffer (0.3% Triton X-100, 1% BSA in 1X PBS) for overnight incubation at 4 °C. Primary antibodies were aspirated, and cells were rinsed three times with 1X PBS for 5 min each wash. Secondary antibodies were applied for two hours at room temperature. Secondary antibodies were then removed and samples rinsed three times with 1X PBS for 5 min each. The cells were then mounted with Vectashield Mounting Medium with DAPI (Vector Labs) and glass coverslips. Images were acquired using an EVOS FL Imaging System (Life Technologies). The following antibodies were used for immunostaining of neurons: MAP2 (Cell Signaling, 4542), TUJ1 (Cell Signaling, 4466), goat anti-rabbit Alexa Fluor 488 (Life Technologies, A-11034), and goat anti-mouse Alexa Fluor 555 (Life Technologies, A-21424).

### Statistical Analysis

All qRT-PCR data represent the results of three biological replicates, analyzed in technical triplicates. TaqMan hPSC Scorecard analysis was performed with one biological replicate per cell line. Biological triplicates were analyzed by alpha-synuclein ELISA. Statistical analysis was performed using SPSS Statistics 22 (IBM). Levene’s homogeneity test was used to evaluate the null hypothesis that population variances were equal. Significance was determined by one-way analysis of variance (ANOVA) with post-hoc Bonferroni where samples met the homogeneity of variances assumption or Games-Howell where samples did not meet the homogeneity of variances assumption.

## Additional Information

**How to cite this article**: Heman-Ackah, S. M. *et al*. Precision Modulation of Neurodegenerative Disease-Related Gene Expression in Human iPSC-Derived Neurons. *Sci. Rep.* **6**, 28420; doi: 10.1038/srep28420 (2016).

## Supplementary Material

Supplementary Information
