# Supplementary material for: Precision Modulation of Neurodegenerative Disease-Related Gene Expression in Human iPSC-Derived Neurons
Source: Sci Rep. 2016 Jun 24;6:28420. doi: 10.1038/srep28420 (PMC4920027; doi:10.1038/srep28420)
Supplement: Supplementary Information [file srep28420-s1.pdf]

Supplementary Information to Accompany

Precision Modulation of Neurodegenerative Disease-Related Gene Expression in Human iPSC-Derived Neurons

Sabrina Mahalia Heman-Ackah[1,2,3,*], Andrew Roger Bassett[4,5,†], Matthew John Andrew Wood[1,5]

[1] Department of Physiology, Anatomy and Genetics, University of Oxford, Oxford, OX1 3QX, UK
[2] NIH Oxford-Cambridge Scholars Program, National Institutes of Health, Bethesda, MD, 20892, US
[3] UNC MD-PhD Program, University of North Carolina at Chapel Hill, Chapel Hill, NC, 27599, US
[4] Genome Engineering Oxford, Sir William Dunn School of Pathology, University of Oxford, Oxford, OX1 3RE, UK
[5] Co-senior author
[†] Current address: Wellcome Trust Sanger Institute, Wellcome Genome Campus, Hinxton, Cambridge, CB10 1SA, UK
[*] Correspondence and requests for materials should be addressed to S.M.H.-A. (e-mail: hemanackah.sabrina@gmail.com)

This PDF file includes:

Supplementary Figure S1
Supplementary Figure S2
Supplementary Table S1
Supplementary Table S2
Supplementary Table S3
Supplementary Methods

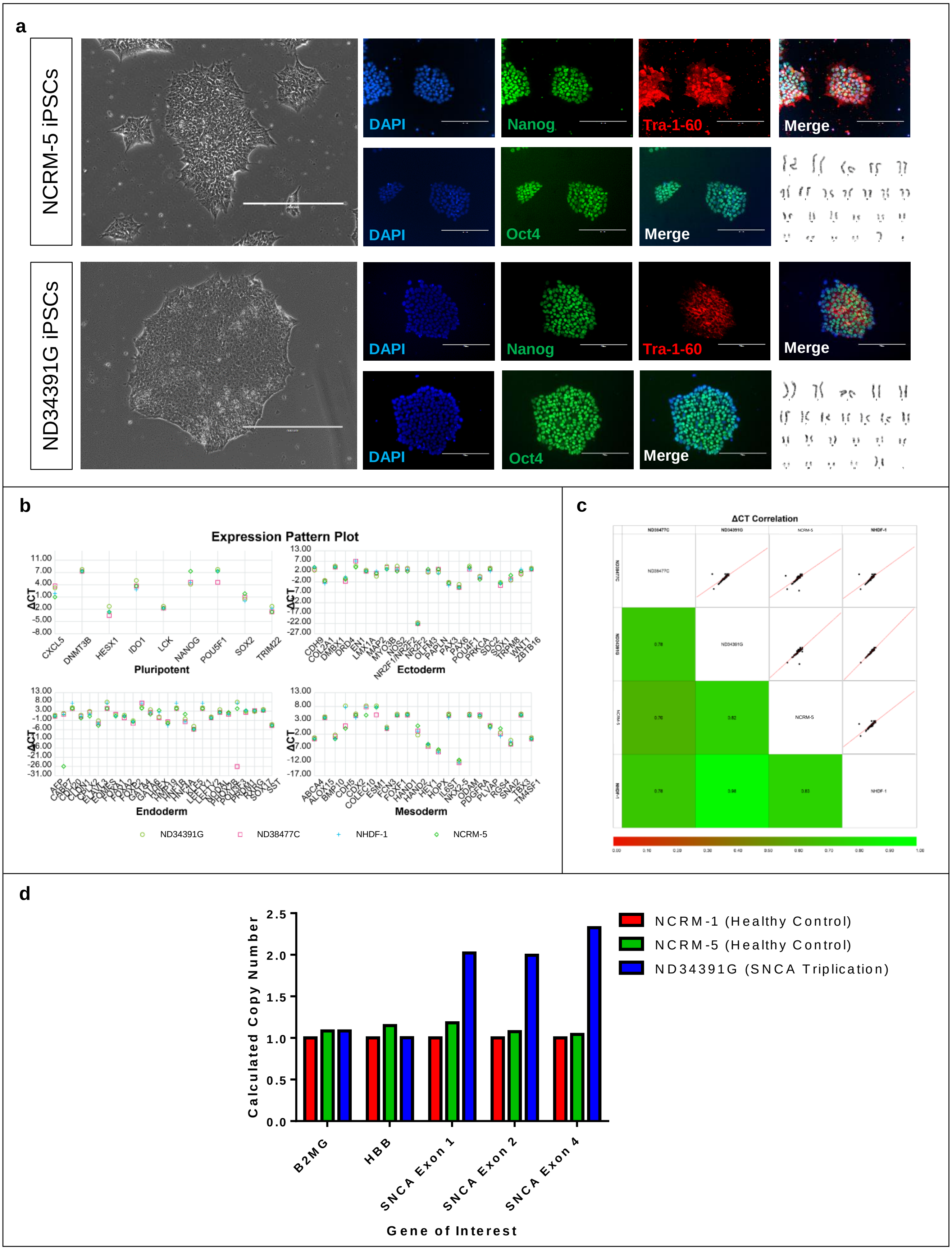

Supplementary Figure S1. iPSC Quality Control

Supplementary Figure S1. iPSC Quality Control

(a) Morphology and pluripotency immunostaining of NCRM-5 (healthy control) and ND34391G (SNCA triplication) iPSCs. Phase images of live iPSCs are shown to the left (scale bars are 400 µm). Upper immunofluorescent micrographs of each cell line show (left to right) DAPI, Nanog, Tra-1-60 and merged images, respectively (scale bars are 200 µm). Lower immunofluorescent micrographs of each cell line show (left to right) DAPI, Oct4, and merged images, respectively (scale bars are 200 µm). To the right of each lower panel are the normal 46,XY and 46,XX g-banded karyotypes of NCRM-5 (healthy control) and ND34391G (SNCA triplication) iPSCs, respectively.

(b) TaqMan hPSC Scorecard gene expression profiling of pluripotency, endodermal, mesodermal and ectodermal fate-specifying genes in NCRM-5 (healthy control) and ND34391G (SNCA triplication) iPSCs compared to other established iPSC lines, ND38477C (PARK2 mutant) and NHDF-1 (healthy control).

(c) Scatterplots and corresponding correlation coefficients of hPSC ScoreCard Ct values for NCRM-5 (healthy control) and ND34391G (SNCA triplication) iPSCs compared to ND38477C (PARK2 mutant) and NHDF-1 (healthy control) iPSCs.

(d) Validation of doubled SNCA copy number in ND34391G (SNCA triplication) iPSCs, compared to NCRM-5 (healthy control) and NCRM-1 (healthy control) iPSCs by Type-it CNV SYBR Green qPCR.

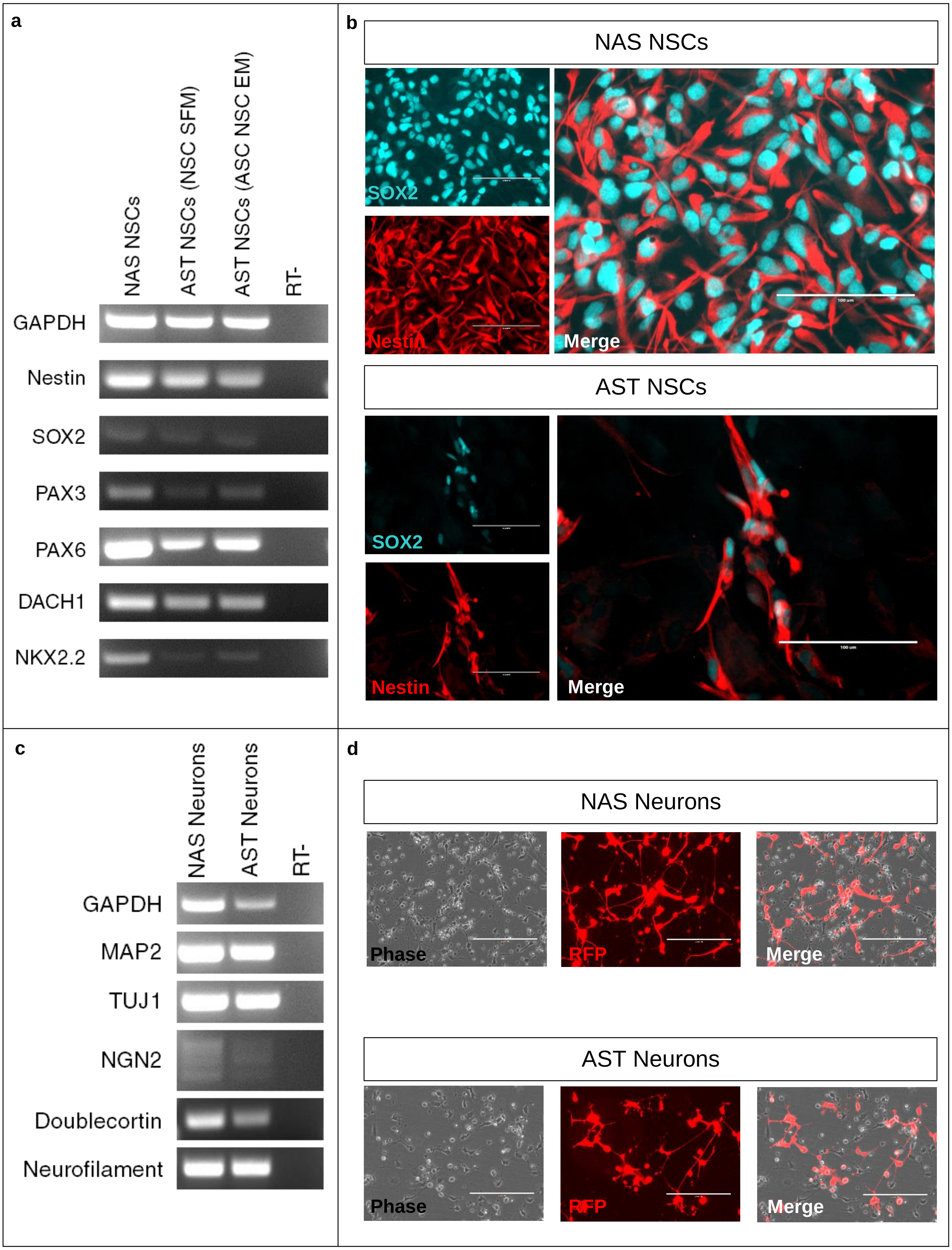

Supplementary Figure S2. Characterization of iPSC-Derived NSCs and Neurons

Supplementary Figure S2. Characterization of iPSC-Derived NSCs and Neurons

(a) RT-PCR expression analysis of NSC markers in NCRM-5, normal alpha-synuclein (NAS) iPSC-derived NSCs and ND34391G, alpha-synuclein triplication (AST) iPSC-derived NSCs. Since AST NSCs were maintained in distinct NSC culture medium (ASC NSC EM), we evaluated whether transitioning them to the same medium as NAS NSCs (NSC SFM) would affect the expression of NSC markers. No such expression alterations were observed. RT- refers to PCR mastermix, including primers, in the absence of cDNA template.

(b) Immunostaining of NAS and AST iPSC-derived NSCs for the NSC markers, SOX2 and Nestin. Scale bars are 100 µm.

(c) RT-PCR expression analysis of neuronal markers in NAS and AST iPSC-derived neurons. RT- refers to PCR mastermix, including primers, in the absence of cDNA template.

(d) NAS and AST iPSC-derived neurons Neon transfected with a CAG-driven tdTomato transfection control plasmid. Scale bars are 200 µm.

Sequence Name	Forward	Reverse
SNCA Exon 1	CACCGCGCACCTCACTTCCGCGTCG	AAACCGACGCGGAAGTGAGGTGCGC
SNCA Exon 1/2 SD	CACCGCCCGCTAACCTGTCGTCGAA	AAACTTCGACGACAGGTTAGCGGGC
SNCA Exon 1/2 SA	CACCGTGAATTCCTTTACACCACAC	AAACGTGTGGTGTAAAGGAATTCAC
SNCA Exon 2	CACCGAGCAGCCACAACTCCCTCCT	AAACAGGAGGGAGTTGTGGCTGCTC
SNCA Exon 4	CACCGTGCTCCCTCCACTGTCTTCT	AAACAGAAGACAGTGGAGGGAGCAC
SNCA TSS1-1	CACCGTCGGAAGATTAGTTAAGCAC	AAACGTGCTTAACTAATCTTCCGAC
SNCA TSS1-2	CACCGAAGCCTTTGCTTTCTGTGCC	AAACGGCACAGAAAGCAAAGGCTTC
SNCA TSS1-3	CACCGTTCCTGGATCACACCAGAA	AAACTTCTGGTGTGATCCAGGAAC
SNCA TSS2-1 (Non-Template)	CACCGCTCCTCCTTCTCCTTCTCCT	AAACAGGAGAAGGAGAAGGAGGAGC
SNCA TSS2-1 (Template)	CACCGCGAGGAGAAGGAGAAGGAGG	AAACCCTCCTTCTCCTTCTCCTCGC
SNCA TSS2-2	CACCGCCCCTCTCTTGGGCCCCTTC	AAACGAAGGGGCCCAAGAGAGGGGC
SNCA TSS2-3	CACCGCACTTCCGCGTCGCGGCGCT	AAACAGCGCCGCGACGCGGAAGTGC
SNCA TSS3-1	CACCGAACCCCGCGCCAGCCACCCG	AAACCGGGTGGCTGGCGCGGGGTTC
SNCA TSS3-2	CACCGCTCAGCTATCTACCCTGAGC	AAACGCTCAGGGTAGATAGCTGAGC
SNCA TSS3-3	CACCGAACAGCAGGCCCAAGTGTGA	AAACTCACACTTGGGCCTGCTGTTC
MAPT TSS-1	CACCGCGAAGAGGGCGCGTTCCTG	AAACCAGGAACGCGCCCTCTTCGC
MAPT TSS-2	CACCGTGGGTGGCGGTGACTGCGA	AAACTCGCAGTCACCGCCACCCAC
MAPT TSS-3	CACCGAGCGGCGCTGCTGTTGGTGC	AAACGCACCAACAGCAGCGCCGCTC
HTT TSS-1	CACCGTTGCGTCCCAGACGCTGCGC	AAACGCGCAGCGTCTGGGACGCAAC
HTT TSS-2	CACCGCGTCCATCTTGGACCCGTCC	AAACGGACGGGTCCAAGATGGACGC
HTT TSS-3	CACCGTGAATGGGGCTCTGGGCCGC	AAACGCGGCCCAGAGCCCCATTCAC
APP TSS1-1	CACCGTAACCCCCAACGTCAAAAGC	AAACGCTTTTGACGTTGGGGGTTAC
APP TSS1-2	CACCGAGTGAAGCTTAAAGGAAAT	AAACATTTCCTTTAAGCTTCACTC
APP TSS1-3	CACCGAGAGGTGGGGCAGGCGTTTC	AAACGAAACGCCTGCCCCACCTCTC
APP TSS2-1	CACCGCTTGAATCATCCGACCCCGC	AAACGCGGGGTCGGATGATTCAAGC

APP TSS2-2 (NGG)	**CACCGAGGTGAGTCCTAGGACGCTG**	**AAACCAGCGTCCTAGGACTCACCTC**
APP TSS2-2 (NAG)	**CACCGCTGAGGCTCTAGAAAAGTC**	**AAACGACTTTTCTAGAGCCTCAGC**
APP TSS2-3	**CACCGAGAGGGACGGTGCAGGATCA**	**AAACTGATCCTGCACCGTCCCTCTC**
APP TSS3-1	**CACCGCGGCGGCGGGGCTCAGAGCC**	**AAACGGCTCTGAGCCCCGCCGCCGC**
APP TSS3-2	**CACCGACCGCTGCCGAGGAAACTGA**	**AAACTCAGTTTCCTCGGCAGCGGTC**
APP TSS3-3	**CACCGCCACCGCCGCCGTCTCCCG**	**AAACCGGGAGACGGCGGCGGTGGC**
APP TSS4-1	**CACCGCCAACTTCTAAGCTAACAA**	**AAACTTGTTAGCTTAGAAGTTGGC**
APP TSS4-2	**CACCGAACTTCTACCACGCACAGCA**	**AAACTGCTGTGCGTGGTAGAAGTTC**
APP TSS4-3	**CACCGCAGAGCTTCCATCCTCGGGA**	**AAACTCCCGAGGATGGAAGCTCTGC**
APP TSS5-1	**CACCGCGTGTTCTCCAAAAAAGAG**	**AAACCTCTTTTTTGGAGAACACGC**
APP TSS5-2	**CACCGCCCACCAGCCTATCCTTCTC**	**AAACGAGAAGGATAGGCTGGTGGGC**
APP TSS5-3	**CACCGTTAGAGATTGGACTTTCAGC**	**AAACGCTGAAAGTCCAATCTCTAAC**

Supplementary Table S1. sgRNA Oligonucleotides

Red: *Bbs* I compatible oligonucleotide overhangs
Underlined: inserted G nucleotides, canonical human U6 transcription start site
Black: sgRNA sequence

Primer Name	Forward	Reverse
ACTB qRT-PCR	CACAGAGCCTCGCCTTTG	GCGGCGATATCATCATCCAT
SNCA qRT-PCR	CCTTCTGCCTTTCCACCCT	TCCCTCCTTGGCCTTTGAAA
SNCA ChIP qPCR	AGATAGGGACGAGGAGCACG	CCCGCACGCACCTCACTT
MAPT qRT-PCR	CCTCGCCTCTGTCGACTATC	CTCTTGGTCTTGGTGCATGG
HTT qRT-PCR	TCAGCTACCAAGAAAGACCGT	TTCCATAGCGATGCCCAGAA
APP qRT-PCR	GGTTTGGCACTGCTCCTG	CAGAACATGGCAATCTGGGG
B2MG qRT-PCR†	CTCACGTCATCCAGCAGAGA	AGTGGGGTGAATTCAGTGT
HBB qRT-PCR†	TTGGACCCAGAGGTTCTTTG	GAGCCAGGCCATCACTAAAG
SNCA Exon 1 qRT-PCR†	AAAGGCCAAGGAGGGAGTT	ATCCTAACCCATCACTCATGAAC
SNCA Exon 2 qRT-PCR	AAGGAGGGAGTTGTGGCTG	AGAACACCCTCTTTTGTCTTTCC
SNCA Exon 4 qRT-PCR†	CCTGTGGATCCTGACAATGA	TGCAAGTTGTCCACGTAATGA
GAPDH RT-PCR‡	ACCACAGTCCATGCCATCAG	TCCACCACCCTGTTGCTGTA
Nestin RT-PCR	GCGGGCTACTGAAAAGTTCC	GCTGAGGGACATCTTGAGGT
SOX2 RT-PCR	AGCATGGAGAAAACCCGGTA	TTTTGCGTGAGTGTGGATGG
PAX3 RT-PCR‡	GAACACGTTCGACAAAAGCA	GCACACAAGCAAATGGAATG
PAX6 RT-PCR‡	AATAACCTGCCTATGCAACCC	AACTTGAACTGGAACTGACACAC
DACH1 RT-PCR‡	GTGGAAAACACCCCTCAGAA	CTTGTTCCACATTGCACACC
NKX2.2 RT-PCR‡	TGCCTCTCCTTCTGAACCTTGG	GCGAAATCTGCCACCAGTTG
MAP2 RT-PCR	CCTTCCTCCATTCTCCCTCC	TCCTGGGATAGCTAGGGGTT
TUJ1 RT-PCR	TGGACATCTCTTCAGGCCTG	ATGATGCGGTCGGGATACTC
NGN2 RT-PCR	CAGGCCAAAGTCACAGCAAC	GGCTCCTCCTCCTCTTCTTC
Doublecortin RT-PCR	ATTGCCTGTGGTCCTGAAAA	CATAGGACCAGGGCTCTTGG
Neurofilament RT-PCR	AGACCCTGGAAATCGAAGCA	TCACGTTGAGGAGGTCTTGG
TSS1 Isoform qRT-PCR	CAGCTCTGAAAGAGTGTGGTGT	TGCCACACCCTGTTTGGTTT
TSS2-1 Isoform qRT-PCR	CATTCGACGACAGTGTGGTG	TGCCACACCCTGTTTGGTTT
TSS2-2 Isoform qRT-PCR	GCAGAGGGACTCAGTGTGGT	TGCCACACCCTGTTTGGTTT
TSS3 Isoform qRT-PCR	TGAACCACACCCCGATGTGG	TGCCACACCCTGTTTGGTTT
Ex 1 TSS1 NTA qRT-PCR	TGTGATCCAGGAACAGCTGT	ACACACGCGAACTTCAAGAC
Ex 1 TSS2-1 NTA qRT-PCR	GAGCGGAGAACTGGGAGTG	GAAAAGGAGCGCACAGGAAG
Ex 1 TSS2-2 NTA qRT-PCR	GAGGAGTCGGAGTTGTGGAG	GATTTCCAAAGACGCCCGTT
Ex 2 NTA qRT-PCR	CAAACAGGGTGTGGCAGAAG	TGAACAAGCACCAAACTGACA
Ex 3 NTA qRT-PCR	AAGGAGGGAGTGGTGCATG	ACTGGGCCACACTAATCACT

Supplementary Table S2. PCR Primers

†From Devine, M. J. *et al.* Parkinson's disease induced pluripotent stem cells with triplication of the alpha-synuclein locus. *Nature communications* **2**, 440, doi:10.1038/ncomms1453 (2011).

‡From Applied StemCell

NTA = nascent transcript analysis

Tissue	p1@SNCA	p4@SNCA	p2@SNCA	p3@SNCA	p5@SNCA	p6@SNCA	p7@SNCA	p9@SNCA	p13@SNCA	p14@SNCA	p11@SNCA
pons_pool1.CNhs10640.10033-101E6	79.932406	9.58373235	13.6619163	12.5744006	9.44779288	1.42736439	0.54375786	1.56330386	0.74766706	0.06796973	0.13593947
temporal_lobe_donor1	43.5892424	7.96319508	15.3041918	13.1761705	8.34961532	3.08819975	1.4659124	0.41741619	0.31546597	0	0.04532766
occipital_lobe_donor1.CNhs11784.10073-102A1	65.7565812	14.3073364	15.4143245	11.145519	8.26080485	6.10271852	2.20761605	0.29811117	0.48849162	0	0.04610483
middle_temporal_gyrus	115.584053	36.776744	13.8258436	6.22162963	3.59471934	3.59471934	1.10606749	0.96780905	0	0	0
cerebellum_adult	70.3582958	7.61169421	3.85809095	3.5471865	2.46485108	0.20909513	0.41819027	0.17223538	0.03685975	0.09851587	0
frontal_lobe_pool1.CNhs10647.10040-101F4	85.1530206	29.5028309	21.7789138	8.35702507	5.38141766	5.63466084	0.37986478	0.31655398	0.25324318	0.12662159	0.0633108
Astrocyte_cerebral_cortex	2.73940055	0	0.59466298	0.4833717	0.93322061	0.095412	0.09124962	0	0	0	0
putamen_donor10196.CNhs12324.10176-103C5	85.9422277	28.5950374	9.26981981	3.77077416	2.35673385	0.78557795	1.88538708	0.94269354	0	0	0
substantia_nigra_donor10252.CNhs12318.10158-103A5	51.0288916	0.8600375	6.73696041	11.8971854	9.03039374	1.00337708	1.29005625	0.57335833	0.14333958	0	0
brain_donor1.CNhs11796.10084-102B3	71.0941527	15.0105853	16.2688624	13.0421726	7.00566172	2.64127723	0.49476319	0.34014969	0.0309227	0.0618454	0.0927681
neuroblastoma_cell_line:CHP-134.CNhs11276.10508-107D4	1.87961824	0.75490974	0.08526291	0.53552421	0.64473385	0	0	0.04263146	0	0	0
medulla_oblongata_adult	37.9362635	3.28738217	5.6916517	6.87921876	5.85174193	0.56672861	0.44997085	0.27651946	0.33160675	0	0.0206988
diencephalon	44.5102447	6.79366893	2.5769089	7.02793337	6.32514003	0.70279334	0.23426445	0.46852889	0	0	0
Neutrophils	14.3473056	0.24966901	1.63105405	0.1381385	0.1381385	1.13534337	0	0	0	0	0
nucleus_accumbens_pool1.CNhs10644.10037-101F1	62.495036	20.4248936	12.3320112	7.06521476	3.72529506	3.46837816	0.44960458	0.89920915	0.19268768	0.06422923	0

corpus_callosum_pool1.CNhs10649.10042-101F6	21.5009614	0.87621473	2.62864419	10.5145767	8.15553709	0.6066102	0.06740113	0.06740113	0.33700566	0	0
pineal_gland_adult	12.5558421	9.97704201	0.84070931	1.4761202	0.33628372	0.26785092	0	0	0	0	0
olfactory_region	160.829743	37.7853011	24.5442981	18.7311749	9.36558745	5.49017195	5.32869631	0.80737823	0.48442694	0	0.16147565
astrocytoma_cell_line:TM-31.CNhs10742.10425-106D2	0	0	0	0	0	0	0	0	0	0	0
amygdala_adult	92.99919	18.1233287	19.8775938	8.68901604	3.88260155	5.02214948	1.9741027	0.35167354	0	0.07033471	0.07033471
pituitary_gland_adult	11.6473797	5.57712261	1.44179376	4.28717043	1.21413157	0.34149329	0.3034828	0.1138311	0	0	0
occipital_cortex_adult	41.0920071	14.1696576	12.7526919	4.25089729	5.66786305	0	0	0	0	0	0
Astrocyte_cerebellum	1.81347645	0	0.17248514	0.38601063	0.31345472	0.04104035	0	0	0.10448491	0	0
hippocampus_adult	103.330534	19.90734	14.7426059	9.06178543	6.62111949	2.87339636	1.07863188	0.9995348	0	0	0
paracentral_gyrus_pool1.CNhs10642.10035-101E8	77.6006847	18.7079109	15.93887	6.55114571	4.99778126	3.91717991	0.33768792	0.67537585	0.13507517	0	0
Neurons	8.70761463	0	1.69042134	4.27426276	3.35939854	0.05583876	0.21264625	0	0	0	0
medial_frontal_gyrus_adult	84.1764771	25.6959772	13.2910227	7.08854544	1.77213636	3.54427272	2.65820454	0.88606818	0	0.88606818	0
medial_temporal_gyrus_adult	101.171077	37.3702393	20.1515527	6.54809102	4.61396092	4.17421033	2.71464215	1.23450365	0	0.06259547	0
dura_mater_donor1.CNhs10648.10041-101F5	19.7773994	1.01098527	3.22251556	1.32691817	0.50549264	0.06318658	0.3159329	0.12637316	0	0.06318658	0.06318658
Meningeal_Cells	1.82413897	0.23158924	0	0.18269705	0.14532002	0	0	0	0	0	0
cerebral_meninges	111.228396	39.0506007	27.64256	7.6784889	6.14279112	8.77541589	3.07139556	0.65815619	0	0.2193854	0.2193854
Neural_stem_cells	3.88873291	0	0	3.38054366	2.07914512	0	0	0	0	0	0

parietal_lobe_adult	69.28882	20.998292	12.833007	9.44064942	5.66328581	1.88777613	1.11230554	0.40104407	0.26768516	0	0.04756186
caudate_nucleus_adult	38.0868796	13.2861208	3.54296554	7.08593109	5.31444831	0.88574139	0.88574139	0.88574139	0	0	0
thalamus_adult	35.8642624	3.62100759	3.98643402	7.45314486	6.98940185	0.51716498	0.77660021	0.25858249	0.0517165	0	0
postcentral_gyrus_pool1.CNhs10638.10032-101E5	66.6278196	18.7452405	12.7599181	5.91954962	3.15709313	2.43359262	0.52618219	0.59195496	0.06577277	0	0.06577277

Supplementary Table S3. CAGE Data

From website: http://fantom.gsc.riken.jp/zenbu/gLyphs/#config=b-IMGb5lG53ntH8qgNeChB;loc=hg19::chr19:36307745..36428760+

Blue: SNCA expression from TSSs identified by CAGE tags in relevant tissues
Pink: Predominant SNCA TSS, as identified by CAGE and validated by our qRT-PCR data

SUPPLEMENTARY METHODS

iPSC Culture

ND34391G (SNCA triplication) iPSCs are distributed through the NINDS Repository Fibroblasts and iPSCs Collection and were obtained as a feeder-based live culture from the Coriell Institute. For feeder-based culture, ND34391G iPSCs were maintained on CF-1 mouse embryonic fibroblasts (Global Stem) in DMEM/F12 (10565-018, Life Technologies) supplemented with 20% KnockOut Serum Replacement (Life Technologies), 1X GlutaMAX (Life Technologies), 1X MEM non-essential amino acids (Life Technologies), 0.1 mM beta mercaptoethanol (Sigma-Aldrich) and 10 ng/mL bFGF (R&D Systems). During feeder-based culture, ND34391G iPSCs were passaged with the StemPro EZPassage tool (Life Technologies) and supplemented with 10 µM ROCK inhibitor (Tocris Bioscience) for the first 24 hours after passaging. For feeder-free adaptation, ND34391G iPSCs were passaged with collagenase IV (Stem Cell Technologies) onto BD Matrigel Matrix Growth Factor Reduced (BD Biosciences)-coated plates in E8 medium (Life Technologies), supplemented with 10 µM ROCK inhibitor for the first 24 hours after passaging.

NCRM-5 (healthy control) and NCRM-1 (healthy control) iPSCs are distributed through RUDCR Infinite Biologics at Rutgers University and were obtained as feeder-free cryostocks from the NIH Center for Regenerative Medicine (NIH CRM). ND38477C (PARK2 mutant) iPSCs are distributed through the NINDS Repository Fibroblasts and iPSCs Collection and were obtained as a feeder-free cryostock from the Coriell Institute. NHDF-1 (healthy control) iPSCs were likewise obtained as a feeder-free cryostock from the James Martin Stem Cell Facility. Cells were thawed onto Matrigel-coated plates in E8 medium, supplemented with 10 µM ROCK inhibitor for the first 24 hours after thawing.

For maintenance of feeder-free cultures, 100% E8 medium was replaced daily. When cells reached 70% confluence, they were passaged with 0.5 mM EDTA (Life Technologies) in calcium- and magnesium-free DPBS (Life Technologies). Cells were seeded at a subcultivation ratio between 1:6 and 1:24 in E8 medium, supplemented with 10 µM ROCK inhibitor for the first 24 hours after passaging.

Pluripotency Immunostaining

Immunostaining was performed as described in the methods section of the main article. The following antibodies were used for pluripotency immunostaining: Nanog (PeproTech, 500-P236), Tra-1-60 (Millipore, MAB4360), Oct4 (Cell Signaling, 2750), goat anti-rabbit

IgG Alexa Fluor 488 (Life Technologies, A-11034) and goat anti-mouse IgM 555 (Life Technologies, A-21426).

TaqMan hPSC Scorecard Analysis

For TaqMan hPSC Scorecard (Life Technologies) analysis, 20 µL of cDNA, made with 1 µg of RNA from each iPSC line (prepared as described in the methods section of the main article), was diluted in 610 µL of nuclease-free water. 630 µL of 2X TaqMan Fast Advanced Master Mix was added and 10 µL of the reaction mixture was loaded per well into the plate, using a fresh tip for each well. Cycling was performed on a StepOnePlus Real-Time PCR System (Life Technologies) under fast cycling conditions with the following program: 50°C for 2 min, 95°C for 20 sec and 40 cycles of 95°C for 1 sec and 60°C for 20 sec. Data were analyzed using hPSC Scorecard Analysis Software (Life Technologies).

CNV Quantification

Quantification of SNCA copy number was performed using Type-IT CNV SYBR Green (Qiagen) qPCR assays, per the manufacturer's instructions. Primers for CNV quantification were designed both within and outside the triplication region. Primer sequences are provided in Supplementary Table S2.

Generation, Maintenance and Characterization of iPSC-derived NSCs

NAS (normal alpha-synuclein) NSCs were derived from NCRM-5 iPSCs by the NIH CRM, using PSC Neural Induction Medium (Life Technologies) per the manufacturer's instructions. NAS NSCs were maintained in StemPro NSC SFM (Life Technologies), containing Knockout DMEM, 1X StemPro Neural Supplement, 1X GlutaMAX, 20 ng/mL EGF and 20 ng/mL bFGF. AST (alpha-synuclein triplication) NSCs were derived from ND34391G iPSCs via the Applied StemCell NSC Generation Service, using the Chambers protocol (Chambers et al. 2009). AST NSCs were maintained in Applied StemCell NSC Expansion Medium, containing 1:1 Neurobasal (Life Technologies) and DMEM/F-12 (11320-033, Life Technologies), 1X B27 supplement (Life Technologies), 1X N2 supplement (Life Technologies), 1X GlutaMAX, and 20 ng/mL bFGF (Peprotech). All NSCs were grown on Geltrex LDEV-Free Reduced Growth Factor Basement Membrane Matrix (Life Technologies)-coated plates, prepared by applying a dilution of 60 µL of Geltrex in 12 mL of DMEM/F12 (11320-074, Life Technologies) at tissue culture dish-dependent coating volumes (i.e. 1 mL per well of 6-well tissue culture dish), and equilibrating at 37°C, 5% CO_2 for one hour prior to cell seeding. Notably, variability

introduced by the derivation of NSCs has been minimized to the extent possible by using protocols based on chemically defined medium for neural induction in adherent culture format. In addition, expression of NSC markers was evaluated in AST NSCs transitioned to StemPro NSC SFM medium, and no alteration in NSC marker expression was detectable (Supplementary Fig. S2). All NSCs were passaged with StemPro Accutase Cell Dissociation Reagent (Life Technologies) and quenched with 5 volumes of medium per 1 volume of Accutase applied. Cells were seeded at a subcultivation ratio between 1:3 and 1:6. NSCs could be maintained for more than 50 passages, while retaining NSC marker expression and neuronal differentiation capacity.

iPSC-derived NSCs were characterized by their expression of NSC markers, including Nestin, SOX2, PAX3, PAX6, DACH1 and NKX2.2 (Supplementary Fig. S2). Primers for RT-PCR of NSC markers are provided in Supplementary Table S2. In addition, iPSC-derived NSCs were co-immunostained with Nestin (BD Biosciences, 611658) and SOX2 (Cell Signaling, 3579P) primary antibodies with goat anti-mouse IgG Alexa Fluor 555 (Life Technologies, A-21424) and goat anti-rabbit IgG Alexa Fluor 488 (Life Technologies, A-11034) secondary antibodies, respectively (Supplementary Fig. S2).
